# Brain MR Image Enhancement for Tumor Segmentation Using 3D U-Net

**DOI:** 10.3390/s21227528

**Published:** 2021-11-12

**Authors:** Faizad Ullah, Shahab U. Ansari, Muhammad Hanif, Mohamed Arselene Ayari, Muhammad Enamul Hoque Chowdhury, Amith Abdullah Khandakar, Muhammad Salman Khan

**Affiliations:** 1 Artificial Intelligence in Healthcare, Intelligent Information Processing Lab, National Center of Artificial Intelligence, University of Engineering and Technology, Peshawar 25120, Pakistan; faizadullah@gmail.com; 2 Faculty of Computer Science and Engineering, Ghulam Ishaq Khan Institute of Engineering Sciences and Technology, Topi 23640, Pakistan; sansari@giki.edu.pk (S.U.A.); muhammad.hanif@giki.edu.pk (M.H.); 3 Technology Innovation and Engineering Education, College of Engineering, Qatar University, Doha 2713, Qatar; arslana@qu.edu.qa; 4 Department of Civil and Architectural Engineering, College of Engineering, Qatar University, Doha 2713, Qatar; 5 Department of Electrical Engineering, College of Engineering, Qatar University, Doha 2713, Qatar; mchowdhury@qu.edu.qa (M.E.H.C.); amitk@qu.edu.qa (A.A.K.); 6 Department of Electrical Engineering (JC), University of Engineering and Technology, Peshawar 24241, Pakistan

**Keywords:** brain tumor segmentation, deep learning, Gibbs ringing artifact, image enhancement, medical image processing

## Abstract

MRI images are visually inspected by domain experts for the analysis and quantification of the tumorous tissues. Due to the large volumetric data, manual reporting on the images is subjective, cumbersome, and error prone. To address these problems, automatic image analysis tools are employed for tumor segmentation and other subsequent statistical analysis. However, prior to the tumor analysis and quantification, an important challenge lies in the pre-processing. In the present study, permutations of different pre-processing methods are comprehensively investigated. In particular, the study focused on Gibbs ringing artifact removal, bias field correction, intensity normalization, and adaptive histogram equalization (AHE). The pre-processed MRI data is then passed onto 3D U-Net for automatic segmentation of brain tumors. The segmentation results demonstrated the best performance with the combination of two techniques, i.e., Gibbs ringing artifact removal and bias-field correction. The proposed technique achieved mean dice score metrics of 0.91, 0.86, and 0.70 for the whole tumor, tumor core, and enhancing tumor, respectively. The testing mean dice scores achieved by the system are 0.90, 0.83, and 0.71 for the whole tumor, core tumor, and enhancing tumor, respectively. The novelty of this work concerns a robust pre-processing sequence for improving the segmentation accuracy of MR images. The proposed method overcame the testing dice scores of the state-of-the-art methods. The results are benchmarked with the existing techniques used in the Brain Tumor Segmentation Challenge (BraTS) 2018 challenge.

## 1. Introduction

Medical imaging plays an important role in disease identification and treatment planning. Generally, Magnetic Resonance Imaging (MRI) and Computed Tomography (CT) are used to monitor the disease progression and diagnosis. However, segmentation of affected regions and identification of disease is sometimes affected by the degraded image quality. Automatic segmentation of cancerous regions in brain MRI is a challenging task [1] due to the presence of noise in MRI generated at the time of acquisition and transmission [2,3], intensity inhomogeneity of MRI [4], variability of intensity ranges due to different vendor scanners, and capturing of non-brain tissues (eyes, spinal cord, and skull) in the brain MRI [5]. In literature, the skull stripping is considered as an important pre-processing step and, therefore, MRI datasets, such as the Multimodal Brain Tumor Segmentation Challenge (BraTS), are already skull stripped and co-registered to T1-contrast enhanced (T1ce) [6]. Moreover, MRI images are usually altered due to the bias field distortion, as a result the intensity of the same tissues varying across the image. In literature, N4ITK proposed by Tustison [7] is frequently employed for the correction of the bias field distortion problems [5]. Furthermore, the brain MRI images are also subjected to noise [2] up to some extent. The noisy MR images affect the subsequent analysis as it becomes difficult to distinguish between normal brain tissues and tumorous region. Hence, different de-noising techniques are suggested in the literature to obtain speckless brain MRI images [8]. Another significant pre-processing step in medical imaging is image registration [9]. Since brain MR images are acquired through different modalities or sequences, image registration is required to transform these images into a common coordinate space system.

In summary, pre-processing techniques such as skull stripping, de-noising, bias field correction, and registration are widely used to prepare brain MRI data for automatic brain tumor segmentation and analysis. Afterwards, the learning models are trained on this pre-processed data [5]. Especially, pre-processing is essential for automatic brain tumor segmentation as it directly influences the performance of deep learning models. This study is focused on investigation of the effects of different pre-processing techniques on the automatic brain MRI segmentation.

### Contribution

The major contributions of this work are as follows:

- A permutation of different pre-processing techniques for brain tumor segmentation is investigated and results are reported.
- It is demonstrated analytically and confirmed experimentally that the Gibbs ringing artifact removal is an important pre-processing technique which improves segmentation performance in brain MRI.
- Finally, a robust pre-processing framework is proposed for the automatic brain tumor segmentation.

## 2. Related Work

In practice, the acquired MRI images consist of both brain and non-brain tissue, i.e., skull, eyes, and spinal cord [5], and cannot be employed directly for the advanced processing. To refine the acquired MRI images, specifically brain MRI images, the commonly used pre-processing steps used in the literature [10,11,12,13,14] are discussed and a detailed literature review on the prevailing pre-processing techniques for MR images is presented in this section.

The presence of skull in brain MRI may affect the learning rate and computational complexity of the learning model. In addition, the presence of eyes and other non-brain tissues in MRI images of the training dataset may lead to misclassification and unexpected results [5]. Therefore, skull stripping is one of the most important pre-processing steps in the brain image analysis as the removal of the skull is expected to reduce the chances of misclassifications [5]. Based on the reported literature [15], skull stripping has a direct impact on the model performance which consequently affects the efficiency in the tumor detection, brain morphometry, and cortical surface reconstruction. Other commonly used methods for the skull stripping are ROBEX [16], BET [17], BSE and BEaST [5], ROBEX [16], and BET [17] which have been tested on the clinical datasets for skull stripping. Another technique known as LABEL [18] has been applied for pediatric brain MRI skull stripping. Moreover, BraTS dataset, one of the publicly available benchmarks for the brain tumor segmentation, is also skull stripped using skull-stripping filter for ITK [19]. Another important pre-processing step for brain image analysis is image registration. Image registration [20,21] is the process of aligning two or more images onto a common coordinate space (anatomical space) that enables images of the same subject to be geometrically aligned with different modalities, sequences, or across time. BraTS dataset MR images are co-registered to the same anatomical template as a pre-processing step. In practice, image registration has been actively applied on the clinical datasets [22,23].

Brain MRI volume might be acquired by different MRI scanners or by the same scanner with different protocols which causes the intensity inhomogeneity problem, i.e., different intensity ranges for the same tissue type. Intensity normalization is the process of scaling the intensities of the image to a reference or standard scale [15]. The method proposed by Nyúl et al. [24] is considered as one of the most widely used methods for the intensity normalization in which linear piecewise mapping of image intensities to a reference scale is employed. Other commonly used techniques in the literature for intensity normalization are data normalization using z-score (zero mean, unit variance) [14], and histogram matching [25].

Quite often, the brain MRI acquisition process is subjected to noises which affect quality of the obtained MRI images, and it becomes difficult to distinguish between the abnormal tissues from the normal ones. Some of the de-nosing methods include anisotropic diffusion filtering (ADF) [26], non-local means (NLM) [27], and independent component analysis (ICA) [28]. ADF is considered as one of the important techniques for de-noising brain MRI which preserves the edges of MRI and improves signal-to-noise ratio of an image. According to Liu et al. [15], noise reduction from the brain MRI is especially important in the context of deep learning framework as the presence of noise negatively impacts the model performance.

Another inherent characteristic of the MRI acquisition process is the magnetic field variation which may also have negative effects (artifacts) on the images [29]. Artifacts are the (undesired) features which show up in the acquired MR image, however, these features do not belong to the original object. These artifacts affect the image quality and cause hurdles in the brain MR image analysis. The artifacts which are caused due to variation in the magnetic field and coil are known as bias field distortion. The most common strategies in the literature to resolve the bias field distortion are the non-parametric non-uniform intensity normalization (N3) [30] and N4ITK [7]. Some other artifacts in MR images might be caused by the MR scanner itself (hardware) or due to the patient’s interaction with hardware [29]. The effects of such artifacts may be eliminated or corrected if the nature and underlying cause of the artifact is known. In this regard, expertise and familiarization with the scanner design and operation plays the key role. Obvious and overt artifacts could be identified and removed/minimized during data acquisition stage or at reconstruction stage if the underlying cause (associated with MR scanning) is known [29].

Kellner et al. [31] proposed a robust technique to remove ringing artifacts from MR images, in which the given image is re-interpolated based on local, subvoxel-shifts to sample the ringing pattern of the image at the zero-crossings of the oscillating sinc-function [31]. Gibbs ringing artifact which is caused by partial Fourier (PF) acquisition and zero filling interpolation in MRI data is thoroughly studied by Lee et al. [32] and a pipeline was developed for Removal of PF-induced Gibbs ringing (RPG) to remove ringing patterns of different periods by applying the conventional method twice. Deep learning based models [33,34] are also employed for Gibbs ringing artifact removal. Maksim et al. [33] proposed an extension of GAS-CNN (Gibbs-ringing Artifact Suppression Convolutional Neural Network) and called it attention-based convolutional neural network for Gibbs-ringing artifact removal. The method proposed by Yida et al. [34] was also employed to reduce Gibbs ringing artifacts in MRI using CNN. The network was trained over two types of images, i.e., with Gibbs ringing artifact images and without Gibbs ringing artifact images. Afterwards, the input images with Gibbs artifacts were refined (Gibbs-free) by the trained network [34]. However, this paper focuses on an important artifact removal technique, named Gibbs ringing artifact removal using local sub-voxel shift technique [31] in association with some other preprocessing techniques to enhance the MR image quality to improve the automatic brain tumor’s segmentation accuracy. The proposed methodology is detailed in the following section.

## 3. Proposed Methodology

This paper focuses on the investigation and analysis of different pre-processing techniques to enquire the impacts of pre-processing techniques separately and jointly on the segmentation performance of the deep learning model. In the first step, Gibbs ringing artifact removal technique is applied and its effects on the segmentation accuracy are studied. To the best of our knowledge, it is the first time that Gibbs ringing artifact removal is used as a pre-processing step for brain MR image analysis using a deep learning model. The detailed discussion about these pre-processing techniques is given in the following sections.

### 3.1. Dataset

BraTS focuses on evaluation of the state-of-the-art methods by providing 3D brain MR images along with ground truth labels annotated by expert physicians for the automatic segmentation of brain tumors [6]. We utilized the BraTS-2018 dataset for the assessment of different pre-processing sequences and identified the better performing one. BraTS data were collected from 19 institutions using various MRI scanners. The training dataset of BraTS-2018 comprises 285 cases (210 High Grade Glioblastomas (HGG) and 75 Low Grade Gliomas (LGG)). Each MR image is of size 240 × 240 × 155, with four 3D MRI modalities (Fluid Attenuated Inversion Recovery (FLAIR), T1, T1c, T2), rigidly aligned, skull stripped, and resampled to 1 × 1 × 1 mm isotropic resolution. There were 3 tumor subregions provided as annotations, such as enhancing tumor, peritumoral edema, and the necrotic and non-enhancing tumor core. These annotations were further combined into 3 nested subregions as whole tumor, tumor core, and enhancing tumor.

### 3.2. Utilized Pre-Processing Techniques

Different combinations of pre-processing techniques were explored for the performance improvement, and it was inferred that bias field distortion correction followed by Gibbs ringing artifact removal of brain MR images offers a robust pre-processing sequence for brain tumor segmentation. The results were benchmarked with the existing top-scoring techniques of the BraTS-2018, BraTS-2015, and BraTS-2013 challenges. To validate the proposed framework, other pre-processing techniques, i.e., intensity normalization, bias field correction, histogram equalization along with Gibbs ringing artifact removal were also employed.

#### 3.2.1. Intensity Normalization

Brain MR images are usually collected from different institutions and hospitals for dataset development [6]. To address the intensity inhomogeneity problem, two different intensity normalization approaches were adopted in this study. The first approach is Nyul intensity normalization [24] and the second approach is Z-score calculation [14]. These techniques are often used for scaling the intensity to a reference intensity scale and hence the intensity normalization of medical imaging is performed [13,14].

#### 3.2.2. Bias Field Correction

As discussed in Section 2, the bias field distortion affects the quality of the acquired MR images. As a result, the intensity of the same tissue may vary across the MR image [5]. In this study, N4ITK [7] approach was employed for the bias field correction which is briefly explained here. Firstly, the image formation model by N3 [30] is used, given as follows:

(1) $$V\left(x\right)=u\left(x\right)f\left(x\right)+n\left(x\right)\;$$

where *V* represents the observed image, *u* is uncorrupted image, *f* is bias field, and *n* denotes noise which is assumed to be the Gaussian and independent. After using the notation *û = log u* and assuming a noise free scenario the model becomes:

(2) $$\hat{v}\left(x\right)=\hat{u}\left(x\right)+\hat{f}\left(x\right)$$

According to Tustison [7], the iterative scheme of N4ITK is given by:

(3) $${\hat{u}}^{n}={\hat{u}}^{n-1}-\underset{{\hat{f}}_{r}^{2}}{\underbrace{S^{*}\left\{{\hat{u}}^{n-1}-E\left[\hat{u}\;\;|{\hat{u}}^{n-1}\;\right]\right\}}}$$

where S* denotes the smoothing factor. According to [7], instead of convergence to the total bias field *f(x)*, an iterative scheme is designed to converge, i.e., ${\hat{f}}_{r}^{n}\to 0$ and calculation of the total bias field is seen by inspecting the nested nature.

(4) $${\hat{u}}^{n}=\hat{v}-\overset{n}{\underset{i=1}{\sum }}{\hat{f}}_{r.}^{i}$$

Thus, the total bias field estimate at the *n*^th^ iteration is the sum of the first *n* residual bias fields, i.e.,

(5) $${\hat{f}}_{e}^{n}=\overset{n}{\underset{i=1}{\sum }}{\hat{f}}_{r.}^{i}$$

**Figure 1:**
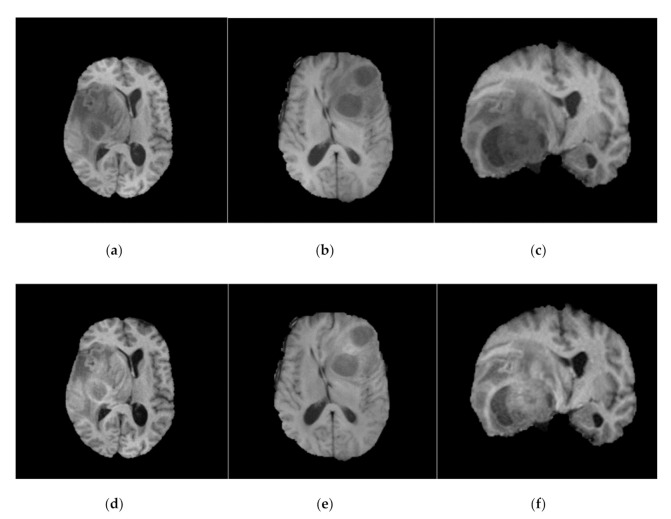
Before (**top row**) and after (**bottom row**) bias field correction. Each MR image in the top row (**a**–**c**) has the corresponding bias field corrected MR image (**d**–**f**) in bottom row. All the images shown here are FLAIR (for fair comparison).

Similar to Kamnitsas et al. [13], N4ITK [7] was employed on BraTS-2013, BraTS-2015, and BraTS-2018 datasets as a pre-processing step. N4ITK [7] is also applied to this work to overcome the bias field distortion and variations in the magnetic field. Sample images from BraTS-2018 dataset before and after bias field correction are shown in Figure 1.

#### 3.2.3. Histogram Equalization

Noise removal and histogram equalization are important and classical image processing techniques to improve the quality of acquired image. In this study, the adaptive histogram equalization (AHE) [35] was used. Initially, 2D slices of 3D volumetric data were stacked to form a block. A histogram of each 2D block was then calculated to enhance the contrast of 3D images. The trilinear interpolation was finally employed to construct the 3d images back. Initially, the global histogram equalization technique was employed, however, it resulted in degradation of the MR image quality visually. Therefore, it is suggested to employ only AHE [35] to achieve the histogram equalized brain MR image.

#### 3.2.4. Gibbs Ringing Artifact Removal

Gibbs ringing artifact is an artifact that degrades the MR image quality, which is also called truncation, spectral leakage, or ringing artifact. It typically appears near sharp high contrast boundaries in the MR images and is also known as Gibbs phenomenon [29]. Gibbs artifact usually occurs at the time of the conversion of MR signals into images using Fourier transform [29]. Skull–brain interface, cerebrospinal fluid (CSF), and spinal cord are more affected by Gibbs ringing artifact in MRI [36]. In this work, Gibbs ringing artifact removal was used as a pre-processing step. To remove the Gibbs artifact from MR image, the local sub-voxel shift [31] technique is employed which is given as follows:

(6) $$j=FT^{-1}\left\{FT\left\{j_{x}\right\}.G_{x}+FT\left\{j_{y}\right\}.G_{y}\right\}$$

where *FT* {.} denotes the Fourier transform, *G_x_* and *G_y_* are two weighting functions, given as:

(7) $$G_{x}=\frac{1+\cos{\left(k_{y}\right)}_{\;}}{\left(1+\cos{\left(k_{y}\right)}_{\;}\right)+\left(1+\cos{\left(k_{x}\right)}_{\;}\right)}$$

(8) $$G_{y}=\frac{1+\cos{\left(k_{x}\right)}_{\;}}{\left(1+\cos{\left(k_{y}\right)}_{\;}\right)+\left(1+\cos{\left(k_{x}\right)}_{\;}\right)}$$

**Figure 2:**
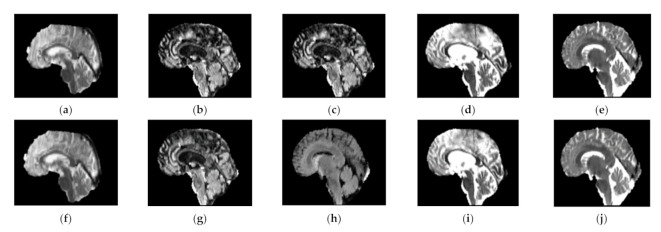
BraTS MR images, before (**top row**) and after (**bottom row**) Gibbs ringing artifact removal. Each MR image in top row (**a**–**e**) has the corresponding Gibbs ringing artifact-removed MR image (**f**–**j**) in the bottom row. All the images shown here are T1ce-saggital (for fair comparison). However, other sequences, i.e., FLAIR, T1 and T2 can also be addressed for ringing artifacts.

Using this approach, the high frequency components along the direction are enhanced and due to the normalization *G_x_* + *G_y_* = 1, where the artifact free images, i.e., *J_x_* = *J_y_* remain unchanged as given in Equation (6), which ensures that the minimal smoothing is introduced [31]. The results produced by Gibbs ringing artifact removal [31] are shown in Figure 2 below.

The top row of Figure 2 shows some sample images from the BraTS 2018 (extension of BraTS 2013 and BraTS 2015) dataset, which exhibits Gibbs ringing artifact. Each image in the top row has a corresponding image in the bottom row, where top row images show that these images are affected by Gibbs ringing artifact and the corresponding bottom row images are Gibbs ringing artifact removed images of the top row. We noticed that a good number of images from the BraTS dataset have the ringing artifact but for the sake of presentation, we added some of the sample images.

### 3.3. Proposed Framework

**Figure 3:**
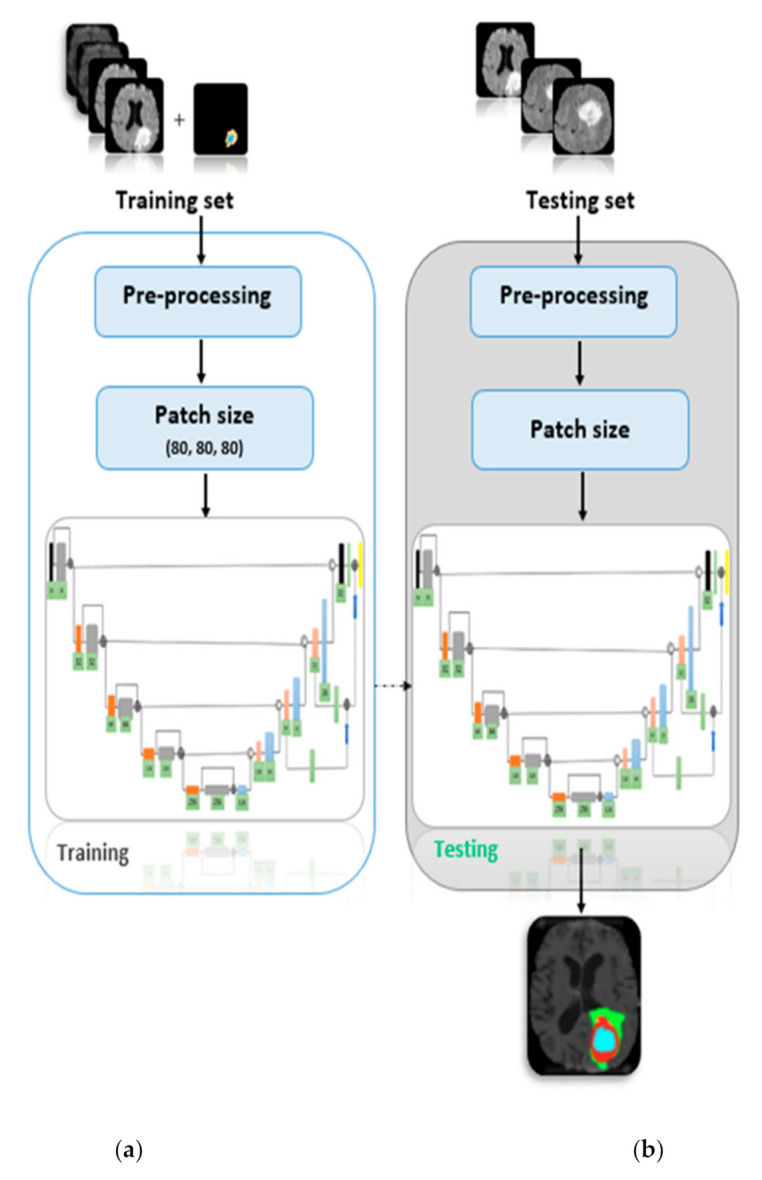
Proposed framework showing the (**a**) training and (**b**) testing model.

The proposed framework is depicted in Figure 3 in which the BraTS (2018, 2015, and 2013) datasets are pre-processed using various pre-processing techniques including bias field correction, intensity normalization, histogram equalization, and Gibbs ringing artifact removal. To train the deep learning model, the U-Net architecture was employed which was originally proposed by Ronneberger [37] for 2D images and further utilized by Isensee et al. [38] for 3D data (3D U-Net). The network (3D U-Net) is identical to Isensee et al. [38] except for the number of epochs, i.e., our network trained over 80 epochs with mini-batch size of 2 and training patch size is 80 × 80 × 80, however, total epochs in Isensee et al. [38] is 300 and the network was trained for a total of 300 epochs. One challenge of medical imaging data is class imbalance as in the BraTS-2017 training dataset there is 166 times as much data label-0 (background) as there is label-1 (enhancing tumor) [18]. Therefore, the multiclass dice loss function was employed to overcome the class imbalance problem as given in Equation (9) below.

(9) $$L_{dc}=-\frac{2}{\left|K\right|}\overset{n}{\underset{k\in K}{\sum }}\frac{\sum_{i}^{n}l_{i,k}m_{i,k}}{\sum_{i}^{n}l_{i,k}+\sum_{i}^{n}m_{i,k}}$$

where *l* is the softmax output of the 3D U-Net and *m* is one hot encoding of the ground truth labels (segmentation maps). Additionally, *i* represents the voxel in training patch and *K* represents the tumor class (i.e., whole tumor, tumor core, and enhancing tumor in BraTS dataset). In Equation (9), *l_i,k_* and *m_i,k_* denote the ground truths and output (softmax output) at voxel *i* of class *k*, respectively, where *i* belongs to *I* (voxel in training patch) and *k* belongs to *K* (one of the tumor classes). The fully convolutional nature of our network, i.e., 3D U-Net, allows the use of arbitrary-size images as we segment the entire patient data at once. In the first stage, several pre-processing techniques were applied in different sequences (separately and jointly). Based on experimental analysis, it was inferred that each pre-processing technique has a significant effect on the segmentation accuracy of brain MRI. However, correcting the brain MRI from bias field distortion (using (4)) followed by Gibbs ringing artifact removal gives us improved and generalized results as shown in (11). Brain MR Images were corrected from bias field using Equation (10).

(10) $${\hat{I}}^{n}=\hat{v}-\overset{n}{\underset{i=1}{\sum }}{\hat{f}}_{r}^{i}$$

In (10), *Î^n^* is a bias field corrected MR image using N4ITK [7]. To remove the Gibbs ringing artifact from brain MR image using local sub-voxel shift [31] technique, Equation (6) was employed. In same sequence, the whole dataset (BraTS-2018) was pre-processed using (11) and network was trained.

(11) $$J=FT^{-1}\left\{FT\left\{{Î}_{x}\right\}.G_{x}+FT\left\{{Î}_{y}\right\}.G_{y}\right\}\;$$

In (11), *FT* {.} denotes the Fourier transform, *Î* denotes a bias field corrected MR image, *G_x_* and *G_y_* are weighting functions as given in (7) and (8), and *J* is the resultant MR image. The complete BraTS-2018 dataset was pre-processed using (10) and (11), i.e., bias field correction followed by Gibbs ringing artifact removal, which we called sequence-6, as there were 10 sequences in total which are discussed in later sections.

## 4. Results and Discussion

In this work, different pre-processing techniques for automatic brain tumor segmentation in brain MR images were evaluated. Experiments were performed on BraTS (2018, 2015 and 2013) datasets and 10 different sequences were evaluated in this work.

### 4.1. Gibbs Artifact Removed vs. Gibbs Not Removed Results

Gibbs ringing artifact removed MR images vs. original images, i.e., the images in BraTS-(2018, 2015, and 2013) were used initially for training the 3D U-Net and results are recorded in Table 1. The purpose of showing the results in Table 1 (Gibbs ranging artifact removed vs. Gibbs ringing artifact not removed) was to examine the importance of Gibbs ringing artifact removal as a pre-processing step. Hence, we can argue that Gibbs removal can be used as a pre-processing step to improve the segmentation accuracy. However, Gibbs removal technique did not improve the core and enhancing region’s accuracy on the BraTS-2013 dataset (Table 1), where we assumed that the size of BraTS-2013 dataset was less than that of BraTS-2015 and BraTS-2018 datasets, generally, the greater size inputs produce the improved results on identical networks.

To confirm and validate the importance of Gibbs ringing artifact removal as a pre-processing technique, original data results (produced by U-Net) compared with pre-processed data, i.e., Gibbs ringing artifact removed along with others pre-processing techniques results, are shown in Table 2. In Table 2, the results produced by Gibbs ringing artifact removed data for training 3D U-Net perform well and overcome the results produced by original/non-removed Gibbs ringing artifacts data. The improved dice score for each column is shown in bold, where we can clearly examine the importance of Gibbs ringing artifact removal as a pre-processing step for training on brain MR images.

### 4.2. Training and Testing Results

The training and testing data split were 80% and 20%, respectively. For results comparison, the standard BraTS metric was used, i.e., dice score. A total of 10 sequences were used for training and testing purposes. All sequence results, i.e., training and testing are shown in Table 3, in which serial no. 1 to 8 results are on the BraTS-2018 dataset while serial no. 9 and serial no. 10 are on the BraTS-2015 and BraTS-2013, respectively. The proposed framework was evaluated on testing data for prediction purposes, where we analyzed that the model is generalized well, as testing results of the model witnessed the generalization of the model as shown in Table 2 and Table 3.

The sequences (order of pre-processing techniques) along with training and testing dice score of each sequence are shown in Table 2, where (✔) and (✖) mean that the corresponding technique was employed to the sequence and not employed to the sequence, respectively, while (✔ *) shows that this technique was employed as pre-processing technique prior to the Gibbs ringing artifact removal technique. In Table 2, Z-score and Nyul means that these two techniques were used for intensity normalization. Intra-sequence results were then compared to analyze the effects of each pre-processing technique. In sequence-1, BraTS-2018 dataset was pre-processed by Gibbs ringing artifact removal and trained using the 3D U-Net. In sequence-2, the BraTS-2018 dataset was pre-processed by two different techniques, i.e., Gibbs ringing artifact removal followed by bias field correction (N4ITK). After that, the 3D U-Net was trained over the data and results were calculated.

In sequence-3, the BraTS-2018 dataset was again pre-processed by two different pre-processing techniques, however, this time AHE replaced the N4ITK [7] technique as a pre-processing technique and results were calculated. In sequence-4 the same data, i.e., BraTS-2018 dataset was pre-processed by three different techniques. This time all three above techniques sequentially pre-processed the data and fed to the network and results were calculated. Sequence-5 comprises two pre-processing techniques, i.e., Gibbs ringing artifact removal and Z-score calculation. In this sequence, after Gibbs ringing artifact removal and Z-score calculation the data was fed to the network to record the results. The most accurate results were produced by the proposed sequence, i.e., sequence-6 in which the Brats-2018 was initially corrected from the bias field distortion using N4ITK [7], and afterwards the Gibbs ringing artifact removal was performed (on bias field corrected data).

The dataset was fed to network and results were recorded after training. Sequence-7 comprises three pre-processing steps, i.e., N4ITK [7], Gibbs ringing artifact removal, and Z-score calculation sequentially. The network was trained on the pre-processed data and results were stored. In sequence-8, the same data, i.e., BraTS-2018 data, was pre-processed sequentially by N4ITK [7], Gibbs ringing artifact removal, and Nyul intensity normalization technique. The network was trained, and results were recorded. BraTS-2015 and BraTS-2013 datasets were also evaluated in this work. In sequence-9 the BraTS-2015 dataset was pre-processed by Gibbs ringing artifact removal and results were calculated after network training. Finally, in sequence-10 the BraTS-2013 dataset was pre-processed by Gibbs-ringing artifact removal and after that the network was trained on the pre-processed data to store the results.

### 4.3. Comparison with Existing Techniques

The proposed methodology (sequence 6), i.e., bias field corrected MRI data followed by Gibbs ringing artifact removal techniques [31] was compared with different published articles on BraTS datasets. The proposed method, i.e., sequence-6 outperformed the state-of-the-art methods on whole and core tumor classes segmentation. However, in enhancing tumor the model did not perform well, roughly it produced the same results as other existing techniques. The enhancing region is generally the region of tumor in brain which lies between other sharpened regions, i.e., whole tumor and core tumor. Therefore, we can assume that this region is affected little by Gibbs ringing artifact while whole region is affected more. We noticed that the training and testing results of the proposed sequence show that the model was generalized well, as the training and testing mean dice scores were close to each other. The results produced by the sequence-6 were compared with the existing techniques in Table 3, where state-of-the-art results were also compared with the existing technique.

### 4.4. Prediction Results

**Figure 4:**
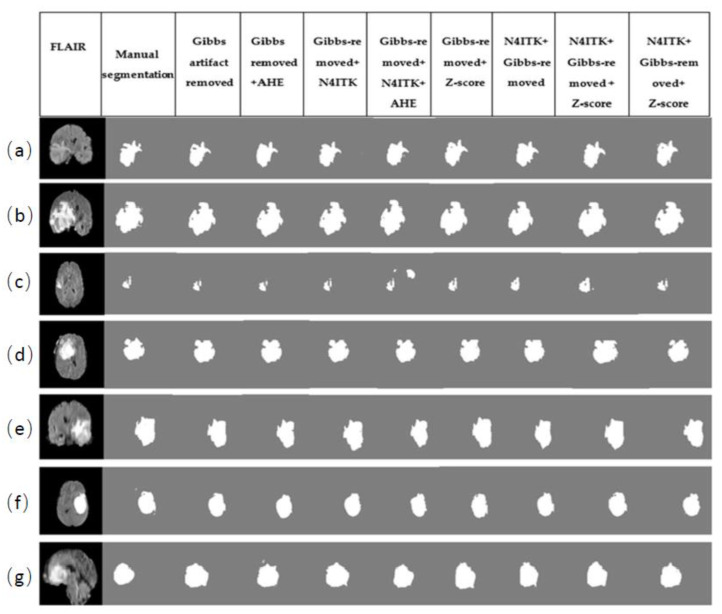
Comparison of prediction (segmentation) results produced by the sequences (1–8) used in this work. Each row (**a**–**g**) has MR images, ground truth labels and their corresponding segmentation results (from left to right).

The permutations of different pre-processing techniques used in this work and their prediction results are depicted in Figure 4. As explained earlier, those 10 different permutations of pre-processing techniques were investigated. For prediction purposes, random images of FLAIR sequence were picked up from the BraTS-2018 dataset. For comparison, BraTS-2018 results were used, i.e., sequence-1 to sequence-8. To confirm the confidence of proposed sequence (sequence 6), the prediction result is shown in Figure 4. FLAIR modality along with the corresponding ground truth label for each patient was segmented via each sequence (1–8). The results show that the sequence-6 has more accurate results produced by other sequences.

## 5. Conclusions and Future Work

Pre-processing the input training data is an essential step for training the deep learning model for automatic brain tumor segmentation. In the present work, different pre-processing techniques, i.e., bias field correction, intensity normalization, histogram equalization, and Gibbs ringing artifact removal were employed to analyze the effect of pre-processing techniques and find out the optimal pre-processing technique (sequence-6) to train the deep network, i.e., 3D U-Net. We also investigated whether bias field correction followed by Gibbs ringing artifact removal gives us improved segmentation results.

To the best of our knowledge, using the Gibbs ringing artifact removal as a pre-processing technique to train the deep learning model was first used by this work. To confirm the confidence of Gibbs ringing artifact removal as a pre-processing technique, Gibbs ringing artifact removal was employed before bias field correction and after bias field correction. It was inferred that the results of Gibbs ringing artifact removal as a preprocessing technique are promising regarding the improvement of segmentation accuracy. Therefore, the use of Gibbs ringing artifact removal is strongly recommended as a pre-processing technique and also segmentation results are improved when Gibbs ringing artifact removal is followed by bias field correction (N4ITK).

It can be noted that Gibbs ringing artifact removal from MR images produced improved results on whole and core regions, however, this technique did not improve the the enhancing region’s accuracy on the BraTS dataset (Table 3). The enhancing region is generally the region of tumor/cancer in the brain which progresses/grows in between whole tumor and core tumor. Therefore, this region may not be affected more by Gibbs ringing artifact as the borders/regions are not sharpened in the enhancing region, because the enhancing region lies between two other sharpened regions of brain. Additionally, all cited works to date have less accuracy on enhancing regions as compared to whole and core tumors.

In this work, the Gibbs ringing artifact removal was applied to brain MR images with other pre-processing techniques to train the model. While, in future, this work can be further extended to apply the Gibbs ringing artifact removal to other parts (organs) of the body acquired with MRI modality.

## Figures and Tables

**Table 1:** Comparison of brain MRI images with (✖) Gibbs ringing artifact and without (✔) Gibbs ringing artifact using local sub voxel-shift method.

No.	BraTS Dataset	Gibbs Ringing Artifact Removed	Dice Score		
			Whole	Core	Enhancing
1	2018	✖	0.88	0.82	**0.67**
2	2018	✔	**0.91**	**0.85**	**0.67**
3	2015	✖	0.85	0.71	**0.57**
4	2015	✔	**0.90**	**0.82**	**0.57**
5	2013	✖	0.84	**0.82**	**0.59**
6	2013	✔	**0.90**	0.81	0.58

**Table 2:** Total sequences investigated in this work along with the training and testing dice scores. Sequence-1 to sequence-8 performed on BraTS-2018 while sequence-9 and sequence-10 performed over BaTS-2015 an BraTS-2013, respectively. * shows that this technique was employed as pre-processing technique prior to the Gibbs ringing artifact removal technique.

Sequence No	Gibbs Artifact Removed	Bias Field Corrected	Intensity Normalized	AHE	Mean Dice Score (Training and Testing)					
					Whole		Core		Enhancing	
					Training	Testing	Training	Testing	Training	Testing
Seq.1	✔	✖	✖	✖	**0.91**	**0.90**	0.85	0.80	0.67	0.60
Seq.2	✔	✔	✖	✖	0.88	0.88	0.83	0.78	0.68	0.57
Seq.3	✔	✖	✖	✔	0.86	0.89	0.75	0.79	0.73	0.59
Seq.4	✔	✔	✖	✔	0.87	0.81	0.73	0.66	**0.64**	0.53
Seq.5	✔	✖	Z-score	✖	0.84	0.86	0.68	0.76	0.63	0.56
Seq.6	✔	✔ *	✖	✖	**0.91**	**0.90**	**0.86**	**0.83**	0.70	**0.71**
Seq.7	✔	✔ *	Z-score	✖	0.81	0.80	0.74	0.68	0.57	0.54
Seq.8	✔	✔ *	Nyul	✖	0.86	0.88	0.78	0.80	0.69	0.56
Seq.9	✔	✖	✖	✖	0.90	0.89	0.82	0.76	0.57	0.53
Seq.10	✔	✖	✖	✖	0.90	0.85	0.81	0.72	0.58	0.52

**Table 3:** Comparison of the proposed methodology with existing techniques.

S. No	Reference	BraTS Dataset	Mean Dice Score (Training)			Mean Dice Score (Testing)		
			Whole	Core	Enhancing	Whole	Core	Enhancing
1	[39]	2013	0.80	0.67	0.85	-	-	-
2	[40]	2015	0.86	**0.86**	0.65	-	-	-
3	[12]	2018	0.86	0.81	0.76	0.84	0.72	0.62
4	[38]	2017	0.89	0.79	0.73	0.85	0.77	0.64
5	[41]	2018	0.89	**0.86**	0.68	0.83	0.78	0.68
6	[13]	2015	0.90	0.75	0.73	0.85	0.67	0.63
7	[42]	2018	0.90	0.81	0.73	0.87	0.79	0.74
8	[43]	2018	0.90	0.83	0.79	-	-	-
9	[11]	2018	0.90	0.84	0.80	0.87	0.79	0.71
10	[44]	2013	-	-	-	0.88	0.79	0.73
11	[23]	2013	-	-	-	0.88	**0.83**	**0.77**
12	[45]	2018	**0.91**	**0.86**	**0.82**	0.88	0.81	0.76
13	Proposed method	2018	**0.91**	**0.86**	0.70	**0.90**	**0.83**	0.71

## Data Availability

MICCAI BraTS 2018 data was used: https://www.med.upenn.edu/sbia/brats2018/data.html (accessed on 7 November 2021).
